# Cytoprotective effects of proteasome β5 subunit overexpression in lens epithelial cells

**Published:** 2007-01-16

**Authors:** Yizhi Liu, Xialin Liu, Tieying Zhang, Coralia Luna, Paloma B. Liton, Pedro Gonzalez

**Affiliations:** 1 Zhongshan Ophthalmic Center of Sun Yat-Sen University, Guangzhou, China; 2 Department of Ophthalmology, Duke University, Durham, NC

## Abstract

**Purpose:**

To determine whether the overexpression of the proteasome catalytic β5 subunit (PSMB5) can induce the expression of the catalytic subunits β1 and β2, enhance proteasome activity, and exert a cytoprotective effect in lens epithelial cells.

**Methods:**

Cells from the human lens epithelial cell line SRA01/04 (LECs) were stably transfected either with a plasmid expressing the proteasome catalytic subunit β5 or with an empty plasmid. β-5-expressing LECs and controls were analyzed for the expression of β1, β2, β5, and α6 proteasome subunits; chymotrypsin-like (CT-L) and peptidylglutamyl-peptide hydrolase (PGPH) catalytic activities; as well as for the accumulation of carbonylated proteins, rates of cell viability, and apoptosis after oxidative stress.

**Results:**

Stable expression of the β5 proteasome subunit resulted in increased expression of the catalytic subunits β1 and β2, increased CT-L and PGPH proteasome activities, and increased resistance to accumulation of carbonylated proteins and cell death after oxidative stress.

**Conclusions:**

The proteasome activity can be genetically "upregulated" in lens cells by overexpression of the β5 catalytic subunit. The resulting increase in proteasome activity leads to a decrease in the accumulation of oxidized proteins and enhanced cell survival following oxidative stress.

## Introduction

The aging process of the eye lens is associated with many posttranslational protein modifications, including the formation of irreversible cross-linked protein aggregates, which are believed to contribute to cataract formation [1-5]. The proteasome is the major cellular proteolytic machinery responsible for the selective elimination of damaged proteins, thus preventing or delaying the accumulation of cross-linked protein aggregates. Lens fibers and lens epithelia have been demonstrated to have a completely functional ubiquitin-proteasome pathway, which can selectively eliminate oxidized proteins [6,7]. An important factor that may contribute to the accumulation of cross-linked protein aggregates is the decrease in proteasome activity observed in aging lenses [8,9]. Although some initial studies have indicated no correlations between proteasome activity and age in human lenses [9], a more broad age range study demonstrated that aging of the human lens is accompanied by a decline in proteasome activity [8]. This age-related decline in proteasome function has been correlated with cataract formation and is thought to contribute to the accumulation of carboxymethylated and ubiquitinated proteins observed in elderly lenses [8]. While this decline in proteasome activity affects preferentially trypsin-like and peptidylglutamyl-peptide hydrolase activities in bovine lenses [10], studies in human lenses showed that the decrease in proteasome function with age is mainly associated with lower levels of peptidylglutamyl-peptide hydrolase activity [8].

Despite the potential importance of enhancing the activity of the proteasome as a means to prevent the accumulation of oxidized proteins and delay cataract formation, there is currently little information about the specific mechanisms by which such an increase in proteasome activity can be accomplished and whether it actually has protective effects in lens cells. Recent results conducted in human fibroblasts have shown that overexpression of just one β-type subunit may be sufficient to increase proteasome assembly and function. Overexpression of either β5 or β1 alone will result in the coordinated overexpression of the other two beta catalytic subunits, and the presence of β-type subunits leads to the recruitment of free (nonintegrated) α subunits and an increased number of proteasomes assembled in the cells [11-15]. The increase in proteasome activity that results from overexpression of single β-type subunit has been shown to exert beneficial effects by delaying cellular senescence and increasing cell survival in response to oxidative stress [13-15].

Given the importance of understanding the role that the age-related decline in proteasome function may have in cataract formation, the objectives of this study were as follows: (1) to determine whether the overexpression of the proteasome catalytic β5 subunit (PSMB5) alone is sufficient to increase the expression of the subunits β1 and β2 and enhance proteasome activity in lens epithelial cells; and (2) to evaluate the potential cytoprotective role of increased proteasome activity in lens epithelial cells by measuring the effects on accumulation of oxidized protein and cell survival following oxidative stress.

## Methods

### Reagents and antibodies

*E. coli* DH5α and the eukaryotic expression plasmid pcDNA-3.1 were obtained from Invitrogen (Carlsbad, CA). Oligonucleotide primers were synthesized by Saibaisheng (Beijing, China). Restriction endonucleases *Eco*RI, *Hin*dIII, and T4 ligase were obtained from TaKaRa Bio Inc. (Osaka, Japan). Calf intestinal alkaline phosphatase was purchased from Sangon Ltd (Shanghai, China). RT-PCR analyses were performed using the One-step RT-PCR Kit (Toyobo Bio-Technology, Shanghai, China). EndoFree Plasmid Maxi kit and lipofection SuperFect were purchased from Qiagen (Valencia, CA). Antibodies against the proteosomal subunits β5 (pw8895), β1 (pw8140), β2 (pw8145), and a-6 (pw8100) were purchased from Affinity Research Products Ltd. (Mamhead Castle, United Kingdom). GAPDH antibody was purchased from Kangcheng (Shanghai,China). The fluorogenic substrates Suc-Leu-Leu-Val-Tyr-AMC (LLVY) and Z-Leu-Leu-Glu-bNA (LLE) were obtained from Bachem AG (Bubendorf, Swithland).

### Human lens epithelial cell line SRA01/04 and culture conditions

Human lens epithelial cells (LEC), line SRA01/04, was a generous gift from Dr. Fu Shang (Tufts university, Boston, MA) [16]. Cells were maintained at a density of 60% confluence in 10 cm dishes at 37 °C in a humidified atmosphere of 5% CO_2_ in Dulbecco's modified eagle medium (DMEM) supplemented with 15% fetal bovine serum (FBS), L-glutamine (2 mM), penicillin (100 units/ml), and streptomycin (0.1 mg/ml). All reagents were obtained from Gibco (Grand Island, NY).

### Construction of the eukaryotic expression plasmid pcDNA3.1-PSMB5

Total RNA was isolated from human LEC (SRA01/04) using TRIzol reagent (Invitrogen Life Technologies). The human PSMB5 coding region sequence was then amplified with the one-step RT-PCR kit (Toyobo Biotechnology, Shanghai, China) using the following specific primers designed according to the PSMB5 sequence gene (NCBI, Unigene Hs.422990). Forward: 5'-GCG AAG CTT ATG GCG CTT GCC AGC GTG T-3', and Reverse: 5'-AGA GAA TTC TCA GGG GGT AGA GCC ACT AT-3', which contain the restriction sites for *Hin*dIII and *Eco*RI, respectively. The PCR amplification conditions were: predenaturation at 94 °C for 4 min, followed by 35 cycles of denaturation at 94 °C for 30 s, annealing at 57 °C for 30 s, polymerization at 68 °C for 50 s at 68 °C, and a last polymerization cycle at 68 °C for 10 min. The PCR product was analyzed in 1% agarose gel, purified, and digested with *Hin*dIII and *Eco*RI. The digested fragment was introduced into the *Hin*dIII/*Eco*RI sites in the eukaryotic expression vector pcDNA3.1(+) by T4 ligation. The obtained pcDNA3.1-PSMB5 plasmid was identified by restriction mapping and further confirmed by DNA sequencing analysis.

### Overexpression of PSMB5 in lens epithelial cells

To overexpress the PSMB5, LECs were transfected with 2 mg of the recombinant plasmid pcDNA3.1-PSMB5 using SuperFect transfection reagent (Qiagen), following the manufacturer's instructions. A pcDNA3.1 empty plasmid was employed as a negative control. Two days after transfection, G418 (700 μg/ml) was added to the culture media for positive selection. Three colonies of stable transfectants were isolated following three to four weeks and propagated into cell lines.

### Immunoblot analysis of proteasomal subunits expression

The expression of β5 subunit as well as several representative 20S proteasomal subunits (β1, β2, and β6) in stably transfected LECs was examined by western blot analysis. Briefly, cell pellets were resuspended in a lysis buffer (50 mM Tris-HCl, 150 mM NaCl, 1% NP-40, 0.1% SDS, and 1 mM PMSF), incubated on ice for 30 min with occasional vortexing, and clarified by centrifugation at 10,000x g for 10 min at 4 °C. Protein concentration was quantified by Bio-Rad Protein Assay (Bio-Rad Laboratories, Hercules, CA). Samples containing equal amounts of protein were resuspended in Laemmli buffer, separated electrophoretically by SDS-PAGE (12% gels), and subsequently transferred to polyvinylidene difluoride (PVDF) membrane. Following transfer, membranes were blocked for 1 h in PBS/Tween (0.01 M PBS with 0.1% Tween-20) containing 5% (w/v) nonfat milk powder. After washing, blots were incubated overnight at 4 °C with the appropriate specific primary antibodies diluted in PBS/Tween containing 5% bovine albumin. The membranes were then washed and incubated in the presence of horseradish peroxidase-conjugated secondary antibodies for 60 min at room temperature. Immunoreactive bands were detected by enhanced chemiluminescence using the ECL plus kit (Amersham, San Francisco, CA).

### Proteasome catalytic activities

Proteasome activity was determined by following the method described by Hosler et al. [17]. Briefly, cells were harvested at 80-90% confluence and centrifuged (400x g for 5 min) into pellets. After three washes with PBS, cell pellets were lysed in proteasome activity buffer (10 mM Tris-HCl, pH 8.0; 1 mM DTT, and 0.2% Nonidet P40) and the concentration adjusted to 1 μg/μl. The proteasomal chymotrypsin-like activity (CT-L) and the peptidylglutamyl-peptide hydrolase activity (PGPH) were determined using the fluorogenic peptide substrates Suc-Leu-Leu-Val-Tyr-AMC (LLVY) and Z-Leu-Leu-Glu-bNA (LLE), respectively. The samples mixed with peptide substrates were incubated at room temperature for 10 min. Fluorescence intensities were monitored at 340 nm excitation and 440 nm emission.

### Protein oxidation analysis

Levels of protein oxidation were determined by spectrophotometric dinitrophenylhydrazone (DNPH) assay, based on the detection of protein carbonyl residues derivatized with 2,4-dinitrophenyl hydrazine (Sigma, St. Louis, MO) as described in Niwa et al. [18]. Cells were lysed in 50 mM Tris-HCl, 150 mM NaCl, 1% NP-40, 0.1% SDS, and 1 mM PMSF. Lysed cells were then clarified by centrifugation at 10,000x g at 4 °C for 10 min and precipitated with cold trichloroacetic acid (TCA, 20% final concentration). A solution of 10 mM DNPH in 2N HCl was added to the protein pellets to yield a final protein concentration of 1-2 mg/ml. Next, 2N HCl was added to blanks. Samples were incubated in the dark at room temperature for 1 h with vortexing every 10 min, followed by precipitation with 10-20% TCA. Protein pellets were washed three times with 1 ml of ethanol/ethyl acetate (1:1, v/v) to remove free DNPH. Samples were then resuspended in 6N guanidine hydrochloride at 37 °C for 15 min by shaking and were measured by spectrophotometry at 370 nm. Carbonyl contents were then calculated in nmol/mg using the formula of Reznick and Packer [19].

### Cell viability assays

Cell viability was determined as a function of mitochondrial activity using 3-[4,5dimethylthiazol-2-yl]-2,5-diphenyltetrazolium bromide substrate (MTT, Sigma). Briefly, human LECs (5x10^4^) stably transfected with either empty vector or pcDNA3.1-PSMB5 plasmid were seeded in 96-well plates and incubated for 24 h. Culture media was removed and cells were then incubated for 4 h in phenol red-free serum-free DMEM (180 μl) containing the appropriate concentration of H_2_O_2_. 20 μl MTT solution (5 mg/ml in PBS) were added to each well, and cells were further incubated for 4 h at 37 °C in 5% CO_2_. After this incubation period, the culture media was replaced with 150 μl of DMSO. The plate was subjected to gentle agitation for 15 min, and the absorbance was read at 570 nm with a reference wavelength of 630 nm. Each experiment was repeated three times. and each data point in each experiment was the average of two readings.

### Apoptosis assays

Apoptosis was assayed by flow cytometry as described in the following references [20,21]. Cells were incubated in serum-free, phenol red-free DMEM in the absence or in the presence of H_2_O_2_ (40 μM H_2_O_2_ was added at single dose) at 37 °C for 24 h. After this period of time, cells were harvested by trypsin digestion, resuspended in one ml of hypotonic propidium iodide (PI) working solution (50 μg/ml PI, 1 g/l sodium citrate, 1 g/l Triton X-100), and incubated overnight at 4 °C. Samples were analyzed in a BD FACS Aria^TM^ system (Becton Dickinson Biosciences, San Jose, CA). In parallel experiments, nuclear damage was visualized by Hoechst 33342 staining [22].

### Statistical analysis

The data was analyzed using a paired Student's t-test. Differences were considered statistically significant only for p values lower than 0.05. All values were expressed as mean±SD.

## Results

### Overexpression of the proteasome β5 subunit in LECs induces the expression of proteasome βsubunit

**Figure 1:**
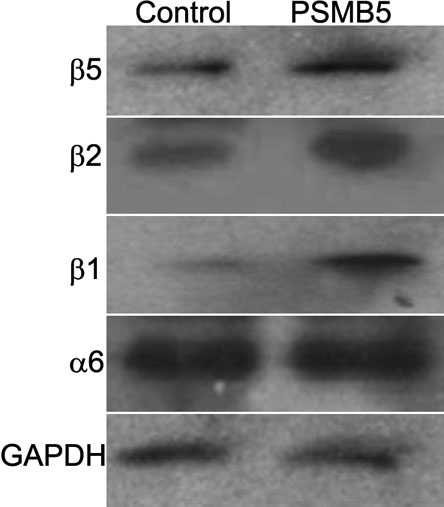
Western blot analysis confirming the overexpression of β5 subunit in LECs stably transfected with PSMB5 plasmid compared to control cells transfected with an empty plasmid. Cells stably transfected with the pcDNA3.1-PSMB5 plasmid also showed increased expression of the catalytic subunits β1 and β2, while expression of the constitutive subunit α6 subunit did not show any significant change. GAPDH was used as a control. This figure is representative of the three independent cell lines analyzed.

Three independent clones transfected with either the pcDNA3.1-PSMB5 or with an empty plasmid were propagated into cell lines. These cell lines did not exhibit any obvious differences in growth rate and morphological characteristics compared to the parental cells. All data and figures presented in the results are representative of all three lines. Overexpression of proteasome β5 subunit in LECs stably transfected with pcDNA3.1-PSMB5 plasmid compared to controls transfected with an empty plasmid was confirmed by western blot analysis (Figure 1). Overexpression of PSMB5 also resulted in a concomitant increase in the expression of the subunits β1 and β2, but did not exert any apparent effect on the expression of the α6 subunit (Figure 1).

### Overexpression of the proteasome β5 subunit in LECs results in an increase of proteasome activity

**Figure 2:**
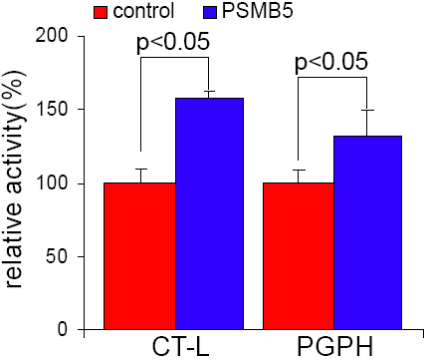
Effects of stable expression of the β5 proteasome subunit on chymotrypsin-like and peptidyl glutamyl-peptide hydrolase catalytic activities in LECs. Mean values of activity were calculated as percentage of activity compared to the control cells. Both proteasome chymotrypsin-like (CT-L) catalytic activity (157.34±6.03%) and proteasome peptidyl glutamyl-peptide hydrolase (PGPH) catalytic activity (132.00±17.11%) were significantly increased in PSMB5 transfected cells as compared with the control cells. Each column shows the average of three independent experiments (%). Similar results were obtained in the three independent cell lines analyzed.

LECs cells overexpressing PSMB5 were used to investigate the effects of the overexpression of this proteasome subunit on the chymotrypsin-like activity (CT-L) of the proteasome, which is mediated by the β5 subunit, as well as on the peptidylglutamyl-peptide hydrolase activity (PGPH), the proteasome activity that shows a larger decrease with age in the lens and is dependent on the catalytic subunit β1. Overexpression of the subunit β5 resulted in a significant increase in both CT-L (157.34±6.03%, p<0.05) and PGPH catalytic activities (132.00±17.11%, p<0.05) as compared with the control cells (Figure 2).

### Overexpression of the proteasome β5 subunit decreases the accumulation of protein oxidization following oxidative stress

**Figure 3:**
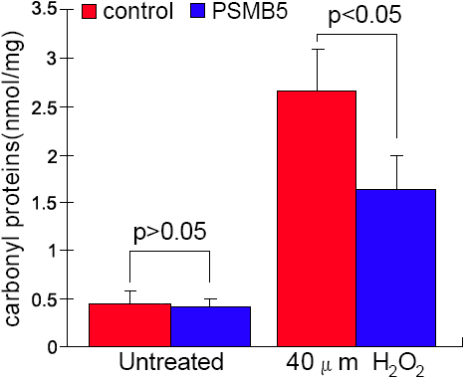
Effects of stable expression of the β5 proteasome subunit on the accumulation of carbonylated proteins after H_2_O_2_ treatment in LECs. Basal level of carbonyl content measured prior to treatment with H_2_O_2_ showed no significant difference between LECs expressing β5 subunit and controls (0.41±0.10 nmol/mg and 0.45±0.12 nmol/mg, respectively, p>0.05). When LECs were exposed to 40 mM H_2_O_2_, carbonyl content increased both in LECs overexpressing β5 and control cells. The accumulation of carbonyl proteins was significantly lower in LECs expressing β5 (1.63±0.36 nmol/mg versus 2.65±0.44 nmol/mg, p<0.01). The result is representative of the three independent cell lines analyzed.

Having established that overexpression of the β5 subunit results in higher proteasome content and proteasome catalytic activity, we investigated whether this "proteasome activity upregulation" could improve the ability of LECs to prevent the accumulation of oxidized proteins after oxidative stress. For this purpose, we treated PSMB5-transfected LECs as well as its counterpart controls with 40 μM H_2_O_2_ and analyzed the presence of carbonyl residues resulting from protein oxidation by spectrophotometric DNPH assay. Generally, carbonyl contents exist in LECs under normal culture conditions [6]. Basal levels of carbonyl content measured prior to treatment with H_2_O_2_ showed no significant difference between LECs expressing PSMB5 and controls (0.41±0.10 nmol/mg and 0.45±0.12 nmol/mg, respectively, p>0.05). When LECs were exposed to 40 μM H_2_O_2_, carbonyl proteins increased dramatically both in LECs expressing PSMB5 and controls. However, this accumulation of oxidized proteins was significantly lower in LECs expressing PSMB5 (1.63±0.36 nmol/mg versus 2.65±0.44 nmol/mg, p=0.036; Figure 3).

### Overexpression of the β5 catalytic subunit in LECs leads to increased survival after oxidative stress

**Figure 4:**
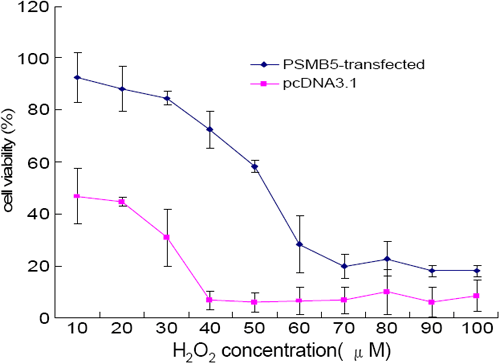
Effects of stable expression of the β5 proteasome subunit on the viability of LECs after H_2_O_2_ treatment. Cell viability was measured by the MTT method 4 h after addition of H_2_O_2_. Treatment of LECs with H_2_O_2_ resulted in a dose-dependent decrease in viability in both β5 expressing cells and nonexpressing controls. Stable expression of β5 resulted in a significant protective effect against H_2_O_2_ toxicity in LECs. Similar results were obtained in the three independent cell lines analyzed.

To evaluate the potential cytoprotective effect of increased proteasome activity, we exposed LECs expressing PSMB5 and parallel controls to different concentrations of H_2_O_2_ ranging from 10 to 100 mM. LEC cultures transfected with PSMB5 showed a dose-dependent decrease in viability that was significantly smaller than that of the control cell cultures, consistent with an increase in the ability to cope with oxidative damage associated with the expression of β5 (Figure 4). The highest levels of differences in cell viability between PSMB5-expressing and control cells were found at concentrations of 40 mM H_2_O_2._

**Figure 5:**
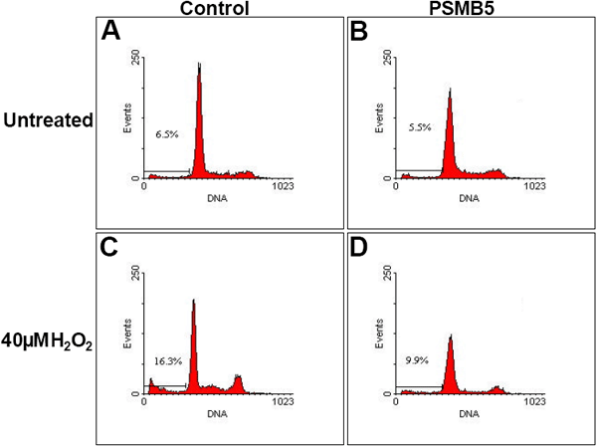
Effects of stable expression of the β5 proteasome subunit on the levels of apoptosis in LECs after H_2_O_2_ treatment. Frequency of apoptotic cells before H_2_O_2_ treatment was 7.1±1.1% for control LECs (**A**) and 5.9±0.8% for LECs expressing β5 (**B**). Twenty-four h after treatment with 40 mM H_2_O_2_, apoptotic cell frequency increased to 15.7±1.9% in control LECs (**C**) while LECs overexpressing the β5 proteasome subunit experienced a smaller rise in apoptosis (9.3±0.9%; **D**). The flow cytometric measurements are representative of three independent experiments.

**Figure 6:**
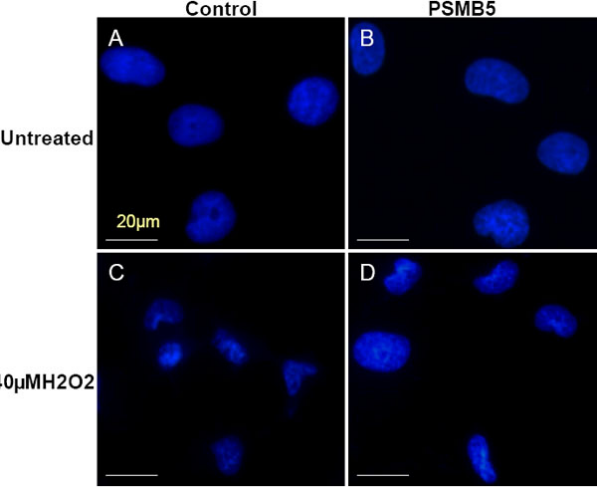
Effects of stable expression of the β5 proteasome subunit on the nuclear morphology of LECs after H_2_O_2_ treatment. Staining with Hoechst 33342 revealed normal nuclear morphology in untreated LECs transfected with an empty plasmid (**A**) or with PSMB5 (**B**). After treatment with 40 mM H_2_O_2_, the nuclei of control LECs showed more shrinking and fragmentation (**D**) than those from PSMB5-transfected cells. Data is representative of three independent experiments.

Expression of PSMB5 also resulted in an increase in resistance to apoptosis after oxidative stress. Levels of apoptosis were measured by flow cytometry with PI staining 24 h after oxidative insult with 40 μM H_2_O_2_. The increase in apoptosis induced by treatment with H_2_O_2_ was significantly lower in the LECs expressing PSMB5 compared to the controls (controls: 15.7±1.9% versus 7.1±1.1%; PSMB5 expressing: 9.3±0.9% versus 5.9±0.8%, p=0.0089; Figure 5). Similar to the apoptotic profile observed by cell sorting, nuclear damage visualized by Hoechst 33342 staining was also more accentuated in the control cells compared to the β5 subunit expressing LECs (Figure 6).

## Discussion

The objective of this study was to evaluate the overexpression of the proteasome subunit β5 in human LECs as a means to increase, experimentally, proteasome function and, in particular, the PGPH activity, which has been shown to decline with age in human lenses [8].

Studies on age-related alterations of proteasome peptidase activities in different tissues have shown a similar decrease of PGPH activity in a variety of tissues [23-29]. The reason for this age-related decrease in PGPH activity in human lens and other tissues is not clear yet. Proteasome subunits, similar to other proteins, may undergo oxidation reactions that result in both structural and functional changes. It has been suggested that preferential decline of PGPH activity with age results from a higher susceptibility of the β1 subunit to inactivation by oxidative processes [30]. Furthermore, cross-linked proteins resulting either from prolonged oxidation or from reactions with lipid peroxidation products, such as 4-hydroxy-2-nonenal, have previously been reported to inhibit the proteasome activity [31-35] and may also contribute to the overall decline in proteasome function in the lens.

The proteasome is believed to play a major role in preventing accumulation of damaged proteins in the lens; therefore identifying the means to modulate proteasome function and revert the age-related decline in PGPH activity should help to assess the relative importance of proteasome function in cataract progression and evaluate the potential therapeutic value for strategies based in proteasome upregulation.

One method that has been proven successful in generating an experimental increase in proteasome activity is the overexpression of the β5 subunit. Overexpression of this subunit in WI38/T and HL60 cells has been shown to elevate the levels of other β-type subunits and increase all three proteasome activities [11,12]. Functional studies have also shown that overexpression of β5 confers enhanced survival following oxidative stress that is associated with decreassed accumulation of oxidized proteins [13-15].

Our results showed that overexpression of the β5 subunit alone was sufficient to induce the expression of the subunits β1 and β2 in LECs. These results are in concordance with what has been reported in human fibroblasts and support that there is a dependent regulation mechanism among the β-type subunits. Although the mechanisms responsible for such coordinated expression of β-subunits are not known, their existence is supported by a number of studies conducted in vertebrate and invertebrate cells [14,15,36-41].

At the functional level, the coordinated expression of β1 and β2 subunits induced by the overexpression of β5 was reflected in the increase in CT-L and PGPH catalytic activities. While CT-L activity is dependent on β5, PGPH activity is associated with the β1 subunit. The observed increase in PGPH activity is particularly relevant, since this is the proteasomal catalytic activity that experiences a more noticeable decline with age in the human lens. Such increase in activity is expected to require the incorporation of β5 subunits into complete proteasomes. Our results did not show any significant effect of β5 overexpression on the levels of constitutive alpha subunits. However, analysis by gel filtration has revealed a higher level of proteasome assembly following expression of β5 subunit in human fibroblasts that is directly linked to the efficient integration of "free" (not integrated) α-type subunits identified to accumulate in the cells [11-15]. There is also evidence that the reported loss of proteasome function upon aging of several human tissues and senescent primary cultures is due to lower levels of β-type subunits, which appear to be "rate-limiting", whereas α-type subunits remain in excess as "free" subunits in senescent cells [11-15]. Therefore, synthesis of additional α subunits may not be needed to assemble new functional proteasomes following the increased expression of β5 and the concomitant increase in expression of β1 and β2 subunits. Other alternative explanations for the observed increase in catalytic activity associated with overexpression of the β5 subunit in absence of a concomitant increase in the expression of α subunits are also possible. For example, some proteasome subunits have been reported to act as free monomers [42,43]. Given the complexity of the proteasome maturation process and its regulation, the specific mechanisms by which overexpression of β-5 might result in an increase of proteasome activity and exert cytoprotective effects will require further investigations to be fully clarified.

The phenotypic effects of overexpression of β5 in lens cells included both: a significant decrease in the accumulation of oxidized proteins and enhanced cell viability and survival after oxidative stress. These results emphasize the importance of proteasome function as a defensive mechanism against oxidative insults and support the concept that the observed decline of proteasome functionality with age may be a relevant contributing factor leading to cataract formation. Accumulation of abnormal proteins in the lens is not only determined by their rates of formation, but also by their rates of elimination. Therefore, the rates of degradation of damaged proteins by the proteasome may constitute a critical factor in crosslinked protein accumulation and cataract formation. Increasing evidence supports the idea that the ability of the proteasome to degrade oxidized proteins serves as a secondary cellular antioxidant defense system [44-47]. Consistent with this notion, once the first line of defense constituted by antioxidant mechanisms, such as the presence of reduced glutathione, is over-run, the maintenance of proper protein structure and configuration in the lens may relay in the ability of the proteasome system to selectively eliminate damaged proteins and prevent their accumulation.

In conclusion, our results suggest that the proteasome can be genetically "upregulated" in lens cells by over expression of the β5 catalytic subunit, and the resulting increase in proteasome activity enhances capacity of the cells to cope with oxidative stress. Because the proteasome is down regulated during the process of aging of the lens, the ability to experimentally increase proteasome activity should be helpful to study the relative importance of the age-related decline of proteasome function in cataract formation and could potentially lead to new therapeutic approaches to delay the progress of this disease.
